# Molecular Surveillance of *Plasmodium* spp. Infection in Neotropical Primates from Bahia and Minas Gerais, Brazil

**DOI:** 10.3390/pathogens14080757

**Published:** 2025-07-31

**Authors:** Luana Karla N. S. S. Santos, Sandy M. Aquino-Teixeira, Sofía Bernal-Valle, Beatriz S. Daltro, Marina Noetzold, Aloma Roberta C. Silva, Denise Anete M. Alvarenga, Luisa B. Silva, Ramon S. Oliveira, Cirilo H. Oliveira, Iago A. Celestino, Maria E. Gonçalves-dos-Santos, Thaynara J. Teixeira, Anaiá P. Sevá, Fabrício S. Campos, Bergmann M. Ribeiro, Paulo M. Roehe, Danilo Simonini-Teixeira, Filipe V. S. Abreu, Cristiana F. A. Brito, George R. Albuquerque

**Affiliations:** 1 Department of Agricultural and Environmental Sciences, Universidade Estadual de Santa Cruz, Ilhéus 45662-900, BA, Brazil; luanakarlanogueira@gmail.com (L.K.N.S.S.S.); sofiabv05@gmail.com (S.B.-V.); bsdaltro.ppgca@uesc.br (B.S.D.); apseva@uesc.br (A.P.S.); dsteixeira@uesc.br (D.S.-T.); 2 Insect Behavior Laboratory, Instituto Federal do Norte de Minas Gerais, Salinas 39600-000, MG, Brazil; sandymicaelly14@gmail.com (S.M.A.-T.); ramonos.oliveira@gmail.com (R.S.O.); cirilohenrique15@gmail.com (C.H.O.); iagoalves958@gmail.com (I.A.C.); dudasantos31082016@gmail.com (M.E.G.-d.-S.); tdjt@aluno.ifnmg.edu.br (T.J.T.); 3 Centro Nacional de Innovaciones Biotecnológicas (CENIBiot), CeNAT, CONARE, San José 1174-1200, Costa Rica; 4 Grupo de Pesquisa em Biologia Molecular e Imunologia da Malária, Instituto René Rachou, Fiocruz, Belo Horizonte 30000-000, MG, Brazil; mnoetzold@aluno.fiocruz.br (M.N.); aloma@fiocruz.br (A.R.C.S.); denise.anete@gmail.com (D.A.M.A.); bs.luisa@outlook.com (L.B.S.); 5 Institute of Basic Health Sciences, Universidade Federal do Rio Grande do Sul, Porto Alegre 90000-000, RS, Brazil; camposvet@gmail.com (F.S.C.); proehe@gmail.com (P.M.R.); 6 Baculovirus Laboratory, Department of Cell Biology, Institute of Biological Sciences, University of Brasilia, Brasília 70000-000, DF, Brazil; bergmann.ribeiro@gmail.com; 7 Laboratory of Hematozoan Transmitting Mosquitoes, Instituto Oswaldo Cruz, Rio de Janeiro 20000-000, RJ, Brazil

**Keywords:** molecular surveillance, *Plasmodium brasilianum*, *Plasmodium malariae*, *Callithrix geoffroyi*, non-human primate reservoirs, zoonotic transmission

## Abstract

In Brazil, *Plasmodium* infections in non-human primates (NHPs) have been associated with *P. simium* and *P. brasilianum*, which are morphologically and genetically similar to the human-infecting species *P. vivax* and *P. malariae*, respectively. Surveillance and monitoring of wild NHPs are crucial for understanding the distribution of these parasites and assessing the risk of zoonotic transmission. This study aimed to detect the presence of *Plasmodium* spp. genetic material in Platyrrhini primates from 47 municipalities in the states of Bahia and Minas Gerais. The animals were captured using Tomahawk-type live traps baited with fruit or immobilized with tranquilizer darts. Free-ranging individuals were chemically restrained via inhalation anesthesia using VetBag^®^ or intramuscular anesthesia injection. Blood samples were collected from the femoral vein. A total of 298 blood and tissue samples were collected from 10 primate species across five genera: *Alouatta caraya* (25), *Alouatta guariba clamitans* (1), *Callicebus melanochir* (1), *Callithrix geoffroyi* (28), *Callithrix jacchus* (4), *Callithrix kuhlii* (31), *Callithrix penicillata* (175), *Callithrix* spp. hybrids (15), *Leontopithecus chrysomelas* (16), *Sapajus robustus* (1), and *Sapajus xanthosthernos* (1). Molecular diagnosis was performed using a nested PCR targeting the *18S small subunit ribosomal RNA* (*18S SSU rRNA*) gene, followed by sequencing. Of the 298 samples analyzed, only one (0.3%) from Bahia tested positive for *Plasmodium brasilianum/P. malariae*. This represents the first detection of this parasite in a free-living *C. geoffroyi* in Brazil. These findings highlight the importance of continued surveillance of *Plasmodium* infections in NHPs to identify regions at risk for zoonotic transmission.

## 1. Introduction

Malaria is a disease caused by protozoan parasites of the genus *Plasmodium*. The infection is transmitted to humans via the bite of female mosquitoes belonging to the genus *Anopheles* [1]. In Brazil, human cases of malaria are endemic in the Legal Amazon Region, which includes states in the Northern Region, as well as Mato Grosso, Tocantins, and Maranhão [2]. However, autochthonous cases have also been reported in areas of the Atlantic Forest (AF). In this biome, the primary vector is *Anopheles* (*Kerteszia*) *cruzi*, commonly known as the bromeliad mosquito. These mosquitoes are of the subgenus *Kerteszia*, typically breed in water-accumulating plants, such as bromeliads, which are endemic to the region. They are frequently implicated in malaria transmission within the AF. The behavioral patterns of *Kerteszia* mosquitoes can vary across geographic areas and seasons, but they are primarily characterized by their feeding habits, which include taking blood meals in the treetops and on the ground, as well as exhibiting vertical dispersal [2,3,4].

Five main species of malaria parasites are known to infect humans: *Plasmodium falciparum*, *Plasmodium vivax*, *Plasmodium ovale* (including the subspecies *Plasmodium ovale* wallikeri and *P. o. curtisi*), *Plasmodium malariae*, and *Plasmodium knowlesi* [5]. In addition to these, approximately 30 *Plasmodium* species are known to infect non-human primates (NHPs), although most of these parasites are restricted to phylogenetically related hosts within the same family [6]. In the Americas, only two *Plasmodium* species are known to naturally infect neotropical NHPs: *Plasmodium brasilianum*, which is genetically and morphologically similar to *P. malariae*, and *Plasmodium simium*, which closely resembles *P. vivax*. Both species have been detected in human infections, highlighting their zoonotic potential [7,8,9].

*Plasmodium brasilianum* has a broad host range, infecting species across all families of neotropical primates, and is distributed from Mexico to southern Brazil. In contrast, *Plasmodium malariae* is a cosmopolitan parasite distributed in sub-Saharan Africa, Southeast Asia, western Pacific islands, and Central and South America [10]. The few molecular epidemiological studies reported so far have shown that *P. brasilianum* and *P. malariae* infections are almost indistinguishable genetically [10]. Sequencing studies of the genes coding for the *circumsporozoite protein* (*csp*) [11], *merozoite surface protein-1* (*msp1*), the *small subunit ribosomal RNA* (*18S rRNA*), and the mitochondrial gene *cytochrome b* (*cytb*) have identified sequences that were 100% identical or had only a few randomly distributed single-nucleotide differences [7,10,11,12,13,14,15,16].

Given the zoonotic potential of *Plasmodium* species infecting neotropical primates and the limited data on their distribution and prevalence in certain regions, this study aimed to assess the presence of *Plasmodium* spp. DNA and to identify the specific *Plasmodium* species in neotropical NHPs from the states of Bahia (BA) and Minas Gerais (MG) in Cerrado (Brazilian tropical savanna), Caatinga (Brazilian dry shrubland), and AF biomes.

## 2. Materials and Methods

### 2.1. Ethical Approval and Permits

All activities were conducted under permits issued by the Biodiversity Authorization and Information System (SISBIO) of the Brazilian Institute of Environment and Natural Resources (ICMBIO) (Nos. 71714-6 and 75734-6) and approved by the Ethics Committee for the Use of Animals of the State University of Santa Cruz (CEUA/UESC) (No. 023/21) and the Ethics Committee of the Federal Institute of Northern Minas Gerais (CEUA/IFNMG) (No. 014/19).

### 2.2. Study Areas

**Figure 1:**
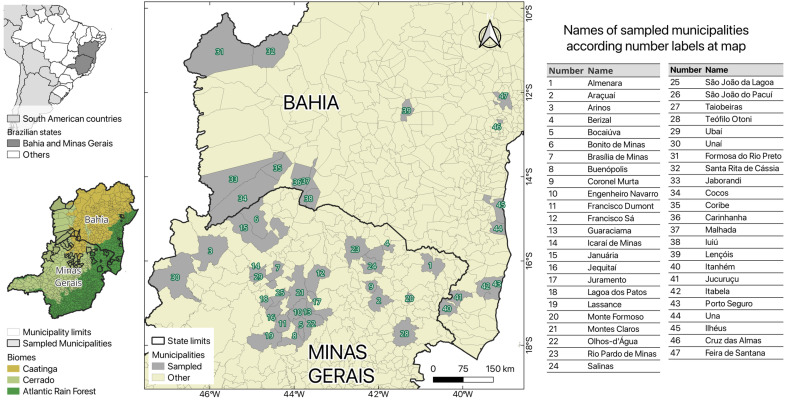
Map with studied areas, in Brazilian states Bahia and Minas Gerais, with their spatial relationship to the biomes.

Seventeen municipalities were selected, distributed throughout the state of Bahia and 30 of Minas Gerais (Figure 1). The sampled municipalities span the three biomes, such as Caatinga, Cerrado, and Atlantic Forest (9.1%, 0.9%, and 0.5% of municipalities in each of them, respectively) and also in ecotone areas (29.6%), with capture areas ranging from forest fragments to rural, peri-urban, and urban environments, both providing context for environmental and ecological factors that may influence *Plasmodium* transmission. These areas were prioritized based on the presence of free-ranging non-human primate populations, identified by the ongoing surveillance programs aimed at monitoring yellow fever. In addition, they represent diverse ecoregions, which may permit the evaluation of different scenarios of *Plasmodium* transmission dynamics.

### 2.3. Capture of NHPs and Sample Collection

Sampling was conducted from August 2019 to December 2023, with field expeditions lasting 5 to 15 days. Capture methods were tailored to the size and behavior of the target species, in which the small animals from the Callitrichidae family and medium-sized species from the Cebidae family were captured using automatic tomahawk traps baited with fruits (e.g., bananas, guavas, papayas), based on local reports of dietary preferences. Trapping sites were georeferenced using GPS, and to attract animals, species-specific vocalizations were played near the traps.

For larger species of the Atelidae family (over 6 kg), anesthetic dart projectors were used, fired by trained professionals using a compressed carbon dioxide rifle with butane gas-propelled darts. Dosages were calculated based on the estimated average weight of males and females of the target species, following guidelines from Brasil [17] and Teixeira et al. [18]. Nets were deployed to cushion the fall of anesthetized primates.

Captured animals were anesthetized using isoflurane (1–2%, dependent effect) delivered via a Vetbag^®^ system with 1.5 L O_2_/min, as recommended by Gamble [19] or ketamine (15 mg/kg) + xilazine (0.5 mg/kg) [17]. Physiological parameters (heart rate, respiratory rate, body temperature, and depth of anesthesia) were monitored every five minutes. The animals were heavy, and blood samples were collected via femoral vein puncture using syringes (for small and large animals, respectively) with and without anticoagulants. The blood was centrifuged at 4000 rpm for 10 min to separate serum and clots. Whole blood, serum, and clots were frozen separately in liquid nitrogen until laboratory analyses. Thick blood smears and Giemsa-stained thin smears were prepared for microscopic examination of *Plasmodium* parasites.

Animals were microchipped to identify recaptures, and they were monitored until full recovery from anesthesia and released at their capture site.

In addition to active captures, tissue samples from deceased NHPs were obtained through surveillance activities in collaboration with local communities and state health departments [19]. Field protocols followed the guidelines by Brasil [17] and Teixeira et al. [18].

### 2.4. DNA Extraction

DNA was extracted from 150 µL of blood clots/whole blood or 10–25 mg of liver tissue (for deceased NHPs) using the QIAamp^®^ DNA Mini Kit (Qiagen, Hilden, Germany). All extractions were performed in a dedicated, contamination-free environment within a biosafety cabinet to minimize the risk of cross-contamination. Clots were macerated and mixed with 300 µL of a lysis buffer, followed by centrifugation at 6000 rpm for 40 s. The protocol was adapted from Abreu et al. [20]. Negative extraction controls were included in each batch to monitor potential contamination.

Fragments of liver (25 mg) and spleen (10 mg) from dead NHPs were used for DNA extraction using the Qiagen Mini Kit (Qiagen, Hilden, Germany) according to the manufacturer’s recommendations. DNA was eluted in a volume of 50 μL of water and stored at 20 °C until use.

### 2.5. Mammalian Cytochrome B PCR

To verify the DNA integrity and suitability for downstream molecular analyses, a fragment of the mammalian *cytochrome b* gene was amplified using primers cyBF (5′-CCCCTCAGAATGATATTTGTCCTCA-3′) and cyBR (5′-CCATCCAACATCTCAGCATGATGAAA-3′), following a protocol adapted from Kocher et al. (1989) [21]. Reactions (20 µL) contained 2 µL of DNA (~1.0 µg), 0.625 µM of each primer, 0.125 mM of dNTPs, 1.875 mM of MgCl_2_, 1U (0.2 µL) of Taq DNA polymerase (Invitrogen), and 1X Standard Taq Reaction Buffer. Cycling conditions were 94 °C for 2 min; 40 cycles of 94 °C for 20 s, 54 °C for 20 s, and 72 °C for 30 s; followed by a final extension at 72 °C for 2 min. Amplicons were separated by electrophoresis on a 2% agarose gel stained with ethidium bromide and visualized under UV light, yielding a 350 bp fragment for neotropical primates.

### 2.6. Nested PCR—18S Target

To detect *Plasmodium* spp. DNA, a nested PCR targeting the *18S small subunit ribosomal RNA* (*18S SSU rRNA*) gene was performed as described by Snounou et al. (1993) [22]. The first reaction used primers rPLU5 (5′-CCTGTTGTTGCCTTAAACTTC-3′) and rPLU6 (5′-TTAAAATTGTTGCAGTTAAAACG-3′) to amplify a 1200 bp genus-specific fragment. The second reaction employed species-specific primers: rFAL1-rFAL2 for *P. falciparum* (205 bp), rVIV1-rVIV2 for *P. vivax* (120 bp), and rMAL1-rMAL2 for *P. malariae* (144 bp). Reactions contained 0.5 µL of each primer (250 µM), along with 10 µL of Promega MasterMix, 2 µL of DNA template, and nuclease-free water to a final volume of 20 µL. Cycling conditions were 95 °C for 5 min; 24 cycles of 94 °C for 1 min, 58 °C for 2 min, and 72 °C for 2 min; followed by a final extension at 72 °C for 5 min. The second reaction used 2 µL of the first reaction product and 29 amplification cycles. The amplicons were separated by electrophoresis on a 2% agarose gel stained with ethidium bromide and visualized under a UV light.

To prevent contamination, all PCR reactions were set up in a designated pre-PCR area, physically separated from the post-PCR workspace. Aerosol-resistant tips were used, and dedicated pipettes were assigned for each step of the process. Negative controls (no-template controls) were included in every reaction to detect potential contamination, while positive controls containing known *Plasmodium* DNA were used to validate the assay.

### 2.7. Nested PCR—Mitochondrial Target and RFLP

To differentiate *P. simium*, a nested PCR targeting the mitochondrial gene *cytochrome oxidase subunit I* (*coxI*) was performed as described by Alvarenga et al. [20]. The first reaction used PsimOutF (5′-CAGGTGGTGTTTTAATGTTATTATCAG-3′) and PsimInR (5′-ATGTAAACAATCCAATAATTGCACC-3′) primers, and the second reaction used PsimEDF (5′-ATCCTACATTTGCTGGAGATCCTA-3′) and PsimEDR (5′-GCTCTTGTATCTACTTCTAAACCTGTAG-3′). The reactions contained 0.5 µL of each primer, along with 10 µL of Promega MasterMix, 2 µL of DNA template, and nuclease-free water to a final volume of 20 µL. Cycling conditions were 94 °C for 2 min; 40 cycles of 94 °C for 30 s, 54 °C for 20 s, and 72 °C for 30 s; followed by a final extension at 72 °C for 2 min. Positive samples were digested with the restriction enzyme *Hpy*CH4III for the RFLP analysis.

### 2.8. Sequencing

Amplified mitochondrial DNA was purified and sequenced using the BigDye^®^ Terminator v3.1 Cycle Sequencing Kit (Thermo Fisher Scientific, Vilnius, Lithuania). The sequence quality was assessed with Chromas Lite v2.1.1, and BLASTn was used to confirm the identity. Sequences were aligned using ClustalW and BioEdit. A phylogenetic analysis was conducted in MEGA X [23] using the Maximum Likelihood method and Tamura–Nei model [24], with branch support evaluated via a bootstrap analysis (1000 replicates).

## 3. Results

**Figure 2:**
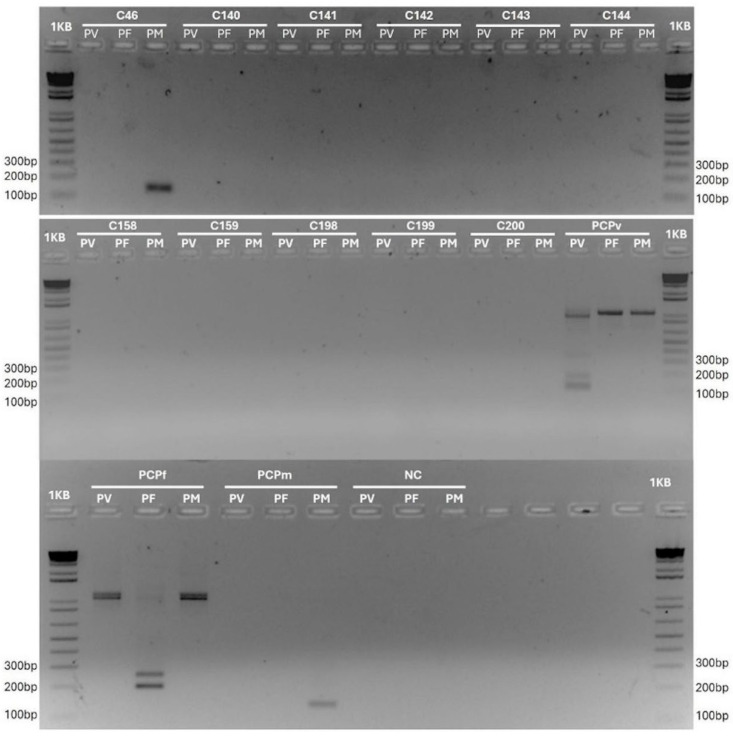
The 2% agarose gel stained with ethidium bromide, showing the amplification of *18S SSU rRNA* from a subset of NHP samples (C46, C140-C144, C158, C159, C198, to C200; see Table S1 for details) using primers for *Plasmodium vivax* (Pv), *Plasmodium falciparum* (Pf), and *Plasmodium malariae* (Pm). The lanes include a 1 kb Molecular Weight Standard Plus DNA Ladder (Invitrogen); positive controls for *P. vivax* (PCPv), *P. falciparum* (PCPf), and *P. malariae* (PCPm); as well as a negative control (NC) with no DNA.

A total of 298 clot/whole blood or liver samples from Platyrrhini primates, collected across 47 municipalities in Minas Gerais (MG) and Bahia (BA), were analyzed (Table S1). The amplification of the mammalian *cytochrome b* gene was successful in all samples, confirming DNA integrity and suitability for molecular analysis. The molecular analysis revealed an overall *Plasmodium* infection rate of 0.34% (1/298; CI 95%: 0.01–0.99%) (Table 1, Figure 2). The positive sample showed the amplification of both the *18S small subunit ribosomal RNA* (*SSU rRNA*) and *cytochrome oxidase subunit I* (*coxI*) genes from *Plasmodium*. All blood smears were negative for *Plasmodium* upon microscopic examination.

**Figure 3:**
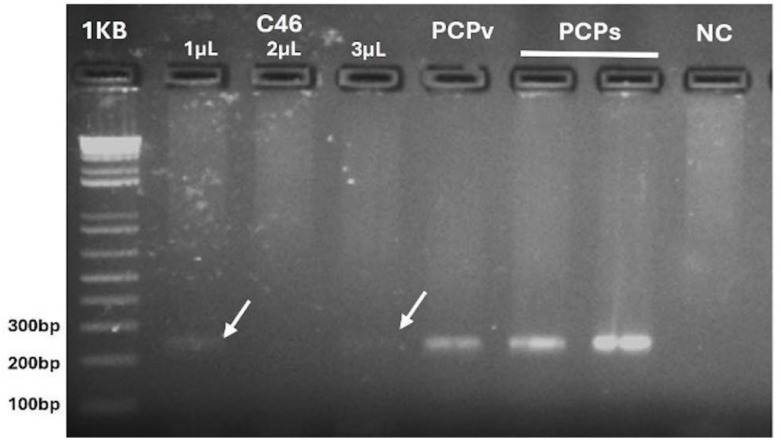
The 2% agarose gel stained with ethidium bromide, showing the amplification of *coxI* [23] from the positive NHP sample (C46). Different volumes of the initial amplification product were used for the reamplification under the same conditions. The gel includes a 1 kb Plus DNA Ladder Molecular Weight Standard (Invitrogen), positive controls for *Plasmodium vivax* (PCPv) and *Plasmodium simium* (PCPs), and a negative control (NC) without DNA.

While the *18S* amplicon fragment failed to produce a viable sequence, the *coxI* amplification product was re-amplified under the same conditions using 1, 2, or 3 µL of their initial reaction (Figure 3).

**Figure 4:**
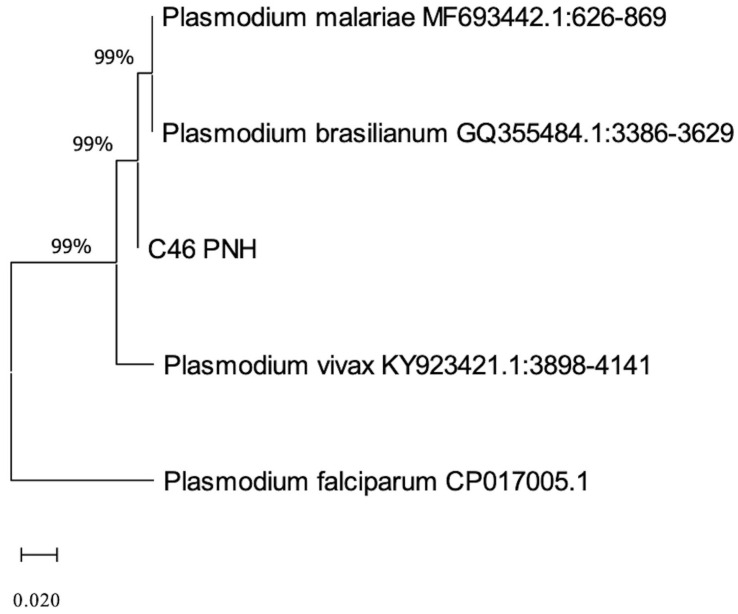
The evolutionary history was inferred by using the Maximum Likelihood method based on the Tamura–Nei model. The tree with the highest log likelihood (-498.95) is shown. Bootstrap support values (1000 replicates) are indicated next to each internal branch. Branch lengths are drawn to scale and represent the number of substitutions per site. The analysis included five nucleotide sequences and 245 aligned positions after complete deletion of gaps and missing data.

The resulting product was successfully sequenced, yielding a high-quality 244 bp sequence. This sequence exhibited 98% identity with *Plasmodium malariae* at sequences available in the GenBank database. Phylogenetic alignment with sequences from various *Plasmodium* species further supported this identification (Figure 4).

Specific PCR followed by genetic sequencing confirmed the presence of *P. brasilianum*/*malariae*. The infected animal was identified as a free-living adult male *Callithrix geoffroyi*, captured in a peri-urban area, surrounding an Atlantic Forest fragment, of the municipality of Porto Seguro, Bahia.

## 4. Discussion

This study represents the largest sampling of NHPs in Minas Gerais and Bahia states for *Plasmodium* infection research and provides the first report of *P. malariae* in free-living *C. geoffroyi.* The low prevalence observed may be influenced by the high number of individuals from the family Callitrichidae, as members of this family generally exhibit a low frequency of *Plasmodium* infections [14,15,25,26,27,28,29]. This low prevalence may be attributed to the natural resistance of Callitrichidae to *Plasmodium* infection [29], as well as their behavior. These primates are highly mobile and have a habit of sleeping in tree hollows from dusk to dawn, which reduces their exposure to mosquito vectors [30]. *Plasmodium* spp. are more frequently detected in genera such as *Alouatta, Ateles, Brachyteles, Cacajao, Cebus,* and *Saimiri* [31].

Despite this, our sampling included 26 specimens from the genus *Alouatta* (family Atelidae), comprising twenty-five *A. caraya* and one *A. guariba clamitans*, all of which tested negative for *Plasmodium*. These specimens were collected from relatively dry regions of the Cerrado or transitional areas between the Atlantic Forest and Cerrado, where bromeliads are scarce. The limited availability of bromeliads likely reduces the presence of the vector *Anopheles* (*Kerteszia*) spp., which may explain the absence of *Plasmodium* in these howler monkeys. Deane [25] previously reported a *P. brasilianum* infection rate of 10.3% (4/39) in *A. caraya*, but all infected individuals were collected in the Amazon-influenced states of Tocantins and Mato Grosso, where anopheline mosquitoes are more prevalent, and also, the human cases are more frequent [32]. Similarly, *P. brasilianum* infection rates ranging from 26% to 70% have been reported in *A. guariba clamitans* [25,33,34] from humid regions of the Atlantic Forest. These regions contrast with the drier collection sites in the present study, where climate and vegetation may not favor the presence of the mosquito vector and, consequently, the parasite.

In this study, *Plasmodium brasilianum*/*P. malariae* DNA was detected in a single *C. geoffroyi* individual—an overweight adult male in good physical condition—captured in an AF area in the district of Trancoso, municipality of Porto Seguro, southern Bahia. This represents the first detection of *P. brasilianum*/*P. malariae* DNA in *C. geoffroyi* in Bahia. Previously, Alvarenga et al. [27] reported the presence of *P. brasilianum*/*P. malariae* in captive *C. geoffroyi* in Rio de Janeiro, also in the AF biome. It is important to emphasize that great care was taken to prevent cross-contamination during all PCR amplifications. In Figure 3, the observation that the 1 µL band was more intense than the 3 µL band, while the 2 µL band was absent, may indicate the presence of inhibitors. The absence of parasites in blood smears suggests low parasitemia, as previously described for Callitrichidae [27].

Porto Seguro is located in the AF biome along the coast of Bahia, a region experiencing significant tourism and rapid urban expansion. The district of Trancoso, historically a fishing village, has undergone a recent population growth and urbanization, leading to deforestation and associated socio-environmental challenges [35]. These environmental changes have broad ecological consequences, impacting both biotic and abiotic factors within the local ecosystem. *Anopheles* (*Kerteszia*) *cruzii*, the primary vector of *Plasmodium* in the AF, thrives in areas with a high bromeliad density [36]. The presence of bromeliads in the region where Callitrichidae were captured suggests favorable conditions for vector proliferation. Furthermore, the Entomological Bulletin of the State of Bahia [37] reported *Anopheles* spp. in 89.4% (373/417) of the municipalities, including Porto Seguro, with the subgenus *Kerteszia* confirmed in the region. There was an outbreak of human malaria in this region in 2021, with fifty-eight confirmed cases of *P. vivax*, seven of them in Porto Seguro [38]. One infected person came from the Amazon, and the pathogen spread in the region.

These findings support the hypothesis that *Plasmodium* circulates in the habitat of the studied primates. Further epidemiological investigations are needed to monitor these animals and clarify the significance of this finding. The presence of infected NHPs in Porto Seguro raises concerns regarding the potential for zoonotic malaria transmission. This underscores the need for enhanced surveillance and public health measures, particularly in tourist areas, to raise awareness among local health authorities and the general public, including residents and visitors, about the potential risk of transmission.

It is still controversial whether *P. brasilianum* and *P. malariae* are genetically indistinguishable. In 2022, Bajic et al. [39] sequenced, for the first time, the complete genome of *P. brasilianum. Plasmodium malariae* and *P. brasilianum* shared 98.4% of nucleotides with a 97.3% pairwise identity. They suggested that both are strains of the same species. However, to understand the degree of similarity between *P. brasilianum* and *P. malariae*, a comprehensive analysis of whole-genome sequencing data using many more parasites from different hosts and different geographic regions is still required.

This study has some limitations that should be considered. First, the sample size, although the largest for NHPs in Minas Gerais and Bahia, may still not fully represent the diversity of NHPs in these regions, as well as the ecoregions that are not included equally, which is a sampling bias. Additionally, the detection method used relies on molecular techniques, which, while highly sensitive, may not detect very low parasitemia levels effectively. The limit of detection for the nested PCR assays used in this study, as described by Snounou et al., 1993 [22] and Alvarenga et al., 2018 [20], is estimated to be between 1 and 5 parasites/µL of blood under optimal conditions. However, this sensitivity can be affected by sample quality, presence of inhibitors, and DNA degradation, particularly in field-collected or stored samples. Therefore, the failure to obtain a sequence from the nested PCR product of the *18S rRNA* gene may be attributed to the presence of inhibitors or to co-infection with multiple *Plasmodium* clones, which can result in overlapping signals and unreadable chromatograms. Future studies with a broader geographical scope, larger sample sizes, and a more integrated approach—including vector surveillance and serological analyses—are necessary to deepen our understanding of *Plasmodium* circulation in NHPs and its potential implications for human health.

## 5. Conclusions

*Plasmodium brasilianum*/*P. malariae* DNA was detected in a blood sample from neotropical NHPs in the state of Bahia, specifically in the AF biome, marking the first report of this parasite in *Callithrix geoffroyi* in the region. The relatively dry climate of the Cerrado appears to limit the transmission of *Plasmodium* spp. among neotropical primates in the studied area, likely due to unfavorable conditions for the vector’s survival and propagation. These findings highlight the importance of continued surveillance to better understand the epidemiology of *Plasmodium* in different ecosystems and its potential implications for both wildlife and human health.

## Figures and Tables

**Table 1:** Molecular detection of *Plasmodium* spp. in non-human primates in Bahia and Minas Gerais, Brazil.

		*Plasmodium* Positives		*Plasmodium* Negatives		
Variable	Category	n	(%)	n	(%)	Total
Gender	Female	0	0.0	142	100.0	144
	Male	1	0.7	155	99.3	156
Living condition	Free-living	1	0.3	291	99.7	292
	Captive	0	0.0	6	100.0	6
Age range	Baby	0	0.0	12	100.0	12
	Juvenile	0	0.0	65	100.0	65
	Adult	1	0.4	220	99.6	221
Species	*Alouatta caraya*	0	0.0	25	100.0	25
	*Alouatta guariba clamitans*	0	0.0	1	100.0	1
	*Callicebus melanochir*	0	0.0	1	100.0	1
	*Callithrix* spp.	0	0.0	15	100.0	15
	*Callithrix geoffroyi*	1	3.6	27	96.4	28
	*Callithrix jacchus*	0	0.0	4	100.0	4
	*Calllithrix khulii*	0	0.0	31	100.0	31
	*Callithrix penicillata*	0	0.0	175	100.0	175
	*Leontopithecus chrysomelas*	0	0.0	16	100.0	16
	*Sapajus robustus*	0	0.0	1	100.0	1
	*Sapajus xanthosthernos*	0	0.0	1	100.0	1
Total	10 species	1	0.3	297	99.7	298

## Data Availability

The data that support the findings of this work are available from the corresponding author upon reasonable request and in Table S1. The sequenced amplicons are available on NCBI.

## Supplementary Materials

The following supporting information can be downloaded at https://www.mdpi.com/article/10.3390/pathogens14080757/s1: Table S1: Overview of Species, Biological Data, and nPCR Results for *Plasmodium* spp. in Neotropical Primates.

## Abbreviations

The following abbreviations are used in this manuscript:

AF	Atlantic Forest
SISBIO	Biodiversity Authorization and Information System
ICMBIO	Brazilian Institute of Environment and Natural Resources
BA	Bahia
MG	Minas Gerais
