# Supplementary material for: Molecular Surveillance of Plasmodium spp. Infection in Neotropical Primates from Bahia and Minas Gerais, Brazil
Source: Pathogens. 2025 Jul 31;14(8):757. doi: 10.3390/pathogens14080757 (PMC12389552; doi:10.3390/pathogens14080757)
Supplement: Supplementary file 1 [file pathogens-14-00757-s001.zip › pathogens-3702505-supplementary.pdf]

State	ID	Specie	Clot	Liver	Spleen	Blood	Municipality	Living condition	Sex	Gender	nPCR *Plasmodium* spp.
Minas Gerais	MG02	*Alouatta caraya*		x			Salinas	Free-living	Male	Juvenile	negative
Minas Gerais	MG03	*Callithrix penicillata*	x				Salinas	Free-living	Female	Baby	negative
Minas Gerais	MG04	*Callithrix penicillata*	x				Salinas	Free-living	Female	Baby	negative
Minas Gerais	MG05	*Callithrix penicillata*	x				Salinas	Free-living	Male	Adult	negative
Minas Gerais	MG06	*Callithrix penicillata*	x				Salinas	Free-living	Male	Adult	negative
Minas Gerais	MG07	*Callithrix penicillata*	x				Salinas	Free-living	Female	Adult	negative
Minas Gerais	MG08	*Callithrix penicillata*	x				Salinas	Free-living	Male	Juvenile	negative
Minas Gerais	MG10	*Callithrix penicillata*	x				Salinas	Free-living	Male	Adult	negative
Minas Gerais	MG11	*Callithrix penicillata*	x				Salinas	Free-living	Male	Adult	negative
Minas Gerais	MG12	*Callithrix penicillata*	x				Salinas	Free-living	Female	Adult	negative
Minas Gerais	MG13	*Callithrix penicillata*	x				Salinas	Free-living	Female	Juvenile	negative
Minas Gerais	MG14	*Callithrix penicillata*	x				Berizal	Free-living	Male	Adult	negative
Minas Gerais	MG15	*Callithrix penicillata*	x				Berizal	Free-living	Male	Adult	negative
Minas Gerais	MG17	*Callithrix penicillata*	x				Berizal	Free-living	Male	Adult	negative
Minas Gerais	MG18	*Callithrix penicillata*	x				Berizal	Free-living	Male	Juvenile	negative
Minas Gerais	MG20	*Callithrix penicillata*	x				Berizal	Free-living	Female	Juvenile	negative
Minas Gerais	MG21	*Callithrix penicillata*		x			Salinas	Free-living	Female	Adult	negative
Minas Gerais	MG22	*Callithrix penicillata*		x			Salinas	Free-living	Female	Juvenile	negative
Minas Gerais	MG23	*Callithrix penicillata*		x			Salinas	Free-living	Male	Adult	negative
Minas Gerais	MG24	*Callithrix penicillata*	x				Salinas	Free-living	Female	Juvenile	negative
Minas Gerais	MG25	*Callithrix penicillata*	x				Salinas	Free-living	Female	Adult	negative
Minas Gerais	MG26	*Callithrix penicillata*	x				Salinas	Free-living	Male	Adult	negative
Minas Gerais	MG30	*Callithrix penicillata*	x				Araçuaí	Free-living	Female	Juvenile	negative
Minas Gerais	MG31	*Callithrix penicillata*	x				Araçuaí	Free-living	Male	Juvenile	negative
Minas Gerais	MG32	*Callithrix geoffroyi*	x				Araçuaí	Free-living	Male	Juvenile	negative
Minas Gerais	MG33	*Callithrix penicillata*	x				Cel. Murta	Free-living	Male	Adult	negative
Minas Gerais	MG34	*Callithrix penicillata*	x				Cel. Murta	Free-living	Female	Adult	negative
Minas Gerais	MG35	*Callithrix penicillata*	x				Cel. Murta	Free-living	Male	Adult	negative

Minas Gerais	MG36	*Callithrix penicillata*	x				Cel. Murta	Free-living	Male	Adult	negative
Minas Gerais	MG37	*Callithrix penicillata*		x			Cel. Murta	Free-living	Male	Juvenile	negative
Minas Gerais	MG38	*Callithrix penicillata*	x				Cel. Murta	Free-living	Male	Adult	negative
Minas Gerais	MG39	*Callithrix penicillata*	x				Cel. Murta	Free-living	Female	Adult	negative
Minas Gerais	MG40	*Callithrix penicillata*	x				Cel. Murta	Free-living	Female	Juvenile	negative
Minas Gerais	MG41	*Alouatta guariba clamitans*		x			Cel. Murta	Free-living	Male	Adult	negative
Minas Gerais	MG42	*Alouatta caraya*	x				Salinas	Free-living	Female	Adult	negative
Minas Gerais	MG43	*Callithrix penicillata*	x				Unaí	Free-living	Female	Adult	negative
Minas Gerais	MG44	*Callithrix penicillata*	x				Unaí	Free-living	Male	Adult	negative
Minas Gerais	MG45	*Callithrix penicillata*	x				Unaí	Free-living	Male	Adult	negative
Minas Gerais	MG46	*Callithrix penicillata*	x				Unaí	Free-living	Female	Adult	negative
Minas Gerais	MG48	*Alouatta caraya*	x				Unaí	Captive	Female	Juvenile	negative
Minas Gerais	MG49	*Alouatta caraya*	x				Unaí	Captive	Male	Adult	negative
Minas Gerais	MG50	*Callithrix penicillata*	x				Arinos	Free-living	Female	Adult	negative
Minas Gerais	MG51	*Callithrix penicillata*	x				Arinos	Free-living	Male	Adult	negative
Minas Gerais	MG52	*Callithrix penicillata*	x				Rio Pardo de Minas	Free-living	Male	Baby	negative
Minas Gerais	MG53	*Callithrix penicillata*	x				Rio Pardo de Minas	Free-living	Female	Adult	negative
Minas Gerais	MG54	*Callithrix penicillata*	x				Rio Pardo de Minas	Free-living	Female	Adult	negative
Minas Gerais	MG55	*Callithrix penicillata*	x				Rio Pardo de Minas	Free-living	Male	Adult	negative
Minas Gerais	MG56	*Callithrix penicillata*				x	Taiobeiras	Free-living	Female	Adult	negative
Minas Gerais	MG57	*Callithrix penicillata*	x				Taiobeiras	Free-living	Female	Adult	negative
Minas Gerais	MG58	*Callithrix penicillata*	x				Taiobeiras	Free-living	Female	Juvenile	negative
Minas Gerais	MG59	*Callithrix penicillata*	x				Taiobeiras	Free-living	Female	Adult	negative
Minas Gerais	MG60	*Callithrix penicillata*	x				Taiobeiras	Free-living	Male	Adult	negative
Minas Gerais	MG61	*Callithrix penicillata*	x				Taiobeiras	Free-living	Male	Adult	negative
Minas Gerais	MG62	*Callithrix penicillata*		x	x		Salinas	Free-living	Female	Adult	negative
Minas Gerais	MG63	*Callithrix penicillata*		x		x	Salinas	Free-living	Female	Adult	negative
Minas Gerais	MG64	*Callithrix penicillata*		x	x		Salinas	Free-living	Male	Adult	negative
Minas Gerais	MG65	*Callithrix penicillata*			x		Icaraí de Minas	Free-living	Female	Adult	negative

Minas Gerais	MG66	*Alouatta caraya*			x		Ubaí	Free-living	Male	Adult	negative
Minas Gerais	MG67	*Callithrix penicillata*			x		Ubaí	Free-living	Female	Adult	negative
Minas Gerais	MG68	*Callithrix penicillata*		x			Ubaí	Free-living	Male	Adult	negative
Minas Gerais	MG69	*Alouatta caraya*	x				Ubaí	Free-living	Female	Adult	negative
Minas Gerais	MG72	*Alouatta caraya*		x	x		Icaraí de Minas	Free-living	Male	Adult	negative
Minas Gerais	MG73	*Alouatta caraya*		x		x	Icaraí de Minas	Free-living	Female	Juvenile	negative
Minas Gerais	MG74	*Alouatta caraya*		x	x		Icaraí de Minas	Free-living	Male	Adult	negative
Minas Gerais	MG75	*Callithrix penicillata*		x			Brasília de Minas	Free-living	Male	Adult	negative
Minas Gerais	MG76	*Alouatta caraya*		x	x		Ubaí	Free-living	Male	Adult	negative
Minas Gerais	MG77	*Alouatta caraya*		x	x		Brasília de Minas	Free-living	Female	Adult	negative
Minas Gerais	MG78	*Callithrix penicillata*	x				Januária	Free-living	Female	Adult	negative
Minas Gerais	MG79	*Callithrix penicillata*	x				Januária	Free-living	Male	Adult	negative
Minas Gerais	MG80	*Alouatta caraya*		x	x		Brasília de Minas	Free-living	Female	Adult	negative
Minas Gerais	MG81	*Alouatta caraya*		x	x		Brasília de Minas	Free-living	Female	Juvenile	negative
Minas Gerais	MG82	*Alouatta caraya*		x	x		Brasília de Minas	Free-living	Female	Juvenile	negative
Minas Gerais	MG83	*Alouatta caraya*		x			Brasília de Minas	Free-living	Female	Juvenile	negative
Minas Gerais	MG84	*Alouatta caraya*		x			Brasília de Minas	Free-living	Female	Adult	negative
Minas Gerais	MG85	*Alouatta caraya*		x	x		Brasília de Minas	Free-living	Male	Adult	negative
Minas Gerais	MG86	*Callithrix penicillata*	x				Brasília de Minas	Free-living	Female	Adult	negative
Minas Gerais	MG87	*Callithrix penicillata*	x				Brasília de Minas	Free-living	Male	Juvenile	negative
Minas Gerais	MG88	*Callithrix penicillata*	x				Brasília de Minas	Free-living	Female	Adult	negative
Minas Gerais	MG89	*Alouatta caraya*		x			Brasília de Minas	Free-living	Female	Juvenile	negative
Minas Gerais	MG90	*Callithrix penicillata*	x				Januária	Free-living	Male	Adult	negative
Minas Gerais	MG91	*Callithrix penicillata*	x				Januária	Free-living	Female	Adult	negative
Minas Gerais	MG92	*Callithrix penicillata*	x				Bonito de Minas	Free-living	Male	Adult	negative
Minas Gerais	MG93	*Callithrix penicillata*	x				Bonito de Minas	Free-living	Female	Adult	negative
Minas Gerais	MG94	*Callithrix penicillata*	x				Bonito de Minas	Free-living	Female	Adult	negative
Minas Gerais	MG95	*Callithrix penicillata*	x				Bonito de Minas	Free-living	Female	Adult	negative
Minas Gerais	MG96	*Callithrix penicillata*		x			Salinas	Free-living	Female	Adult	negative

Minas Gerais	MG97	*Callithrix penicillata*	x				Salinas	Free-living	Male	Adult	negative
Minas Gerais	MG98	*Callithrix penicillata*	x				Salinas	Free-living	Female	Juvenile	negative
Minas Gerais	MG99	*Callithrix penicillata*	x				Salinas	Free-living	Female	Adult	negative
Minas Gerais	MG100	*Callithrix penicillata*	x				Salinas	Free-living	Male	Adult	negative
Minas Gerais	MG101	*Callithrix penicillata*	x				Salinas	Free-living	Male	Adult	negative
Minas Gerais	MG102	*Callithrix penicillata*		x			Francisco Dumont	Free-living	Male	Adult	negative
Minas Gerais	MG102	*Callithrix penicillata*			x		Francisco Dumont	Free-living	Male	Adult	negative
Minas Gerais	MG103	*Callithrix penicillata*		x			Bocaiúva	Free-living	Male	Adult	negative
Minas Gerais	MG103	*Callithrix penicillata*			x		Bocaiúva	Free-living	Male	Adult	negative
Minas Gerais	MG104	*Callithrix penicillata*		x			Jequitaí	Free-living	Male	Baby	negative
Minas Gerais	MG105	*Callithrix penicillata*		x			São João da Lagoa	Free-living	Male	Juvenile	negative
Minas Gerais	MG106	*Callithrix penicillata*		x			São João da Lagoa	Free-living	Male	Juvenile	negative
Minas Gerais	MG108	*Callithrix penicillata*	x				Salinas	Free-living	Female	Baby	negative
Minas Gerais	MG110	*Callithrix penicillata*	x				Salinas	Free-living	Male	Adult	negative
Minas Gerais	MG111	*Callithrix penicillata*	x				Buenópolis	Free-living	Female	Adult	negative
Minas Gerais	MG112	*Callithrix penicillata*	x				Buenópolis	Free-living	Male	Adult	negative
Minas Gerais	MG113	*Callithrix penicillata*	x				Buenópolis	Free-living	Female	Adult	negative
Minas Gerais	MG114	*Callithrix penicillata*		x			Salinas	Free-living	Female	Adult	negative
Minas Gerais	MG115	*Callithrix penicillata*		x			Salinas	Free-living	Male	Adult	negative
Minas Gerais	MG116	*Callithrix penicillata*		x			Ubaí	Free-living	Male	Juvenile	negative
Minas Gerais	MG117	*Callithrix penicillata*		x			Ubaí	Free-living	Female	Adult	negative
Minas Gerais	MG118	*Callithrix penicillata*		x			Ubaí	Free-living	Male	Juvenile	negative
Minas Gerais	MG120	*Callithrix penicillata*		x			Montes Claros	Free-living	Female	Adult	negative
Minas Gerais	MG121	*Callithrix penicillata*		x			Guaraciama	Free-living	Female	Juvenile	negative
Minas Gerais	MG122	*Callithrix penicillata*		x			Olhos D'água	Free-living	Male	Adult	negative
Minas Gerais	MG123	*Callithrix penicillata*		x			Lagos dos Patos	Free-living	Male	Juvenile	negative
Minas Gerais	MG124	*Callithrix penicillata*		x			Engenheiro Navarro	Free-living	Female	Adult	negative
Minas Gerais	MG125	*Callithrix penicillata*		x			Lassance	Free-living	Male	Juvenile	negative
Minas Gerais	MG126	*Callithrix penicillata*		x			Montes Claros	Free-living	Male	Juvenile	negative

Minas Gerais	MG127	*Callithrix penicillata*		x			Francisco Dumont	Free-living	Female	Juvenile	negative
Minas Gerais	MG128	*Callithrix penicillata*		x			Francisco Dumont	Free-living	Female	Adult	negative
Minas Gerais	MG129	*Callithrix penicillata*		x			Francisco Dumont	Free-living	Female	Adult	negative
Minas Gerais	MG130	*Callithrix penicillata*		x			Montes Claros	Free-living	Male	Juvenile	negative
Minas Gerais	MG132	*Callithrix penicillata*		x			Salinas	Free-living	Male	Adult	negative
Minas Gerais	MG133	*Callithrix penicillata*		x			Salinas	Free-living	Male	Adult	negative
Minas Gerais	MG134	*Callithrix penicillata*		x			Bonito de Minas	Free-living	Female	Adult	negative
Minas Gerais	MG135	*Callithrix penicillata*		x			Bonito de Minas	Free-living	Female	Adult	negative
Minas Gerais	MG136	*Alouatta caraya*	x	x	x	x	Salinas	Free-living	Male	Adult	negative
Minas Gerais	MG137	*Callithrix penicillata*		x			Ubaí	Free-living	Male	Adult	negative
Minas Gerais	MG138	*Callithrix penicillata*		x			Ubaí	Free-living	Male	Adult	negative
Minas Gerais	MG139	*Callithrix penicillata*		x			Ubaí	Free-living	Male	Adult	negative
Minas Gerais	MG140	*Callithrix penicillata*		x			Ubaí	Free-living	Female	Adult	negative
Minas Gerais	MG141	*Callithrix penicillata*		x			Montes Claros	Free-living	Female	Juvenile	negative
Minas Gerais	MG142	*Callithrix penicillata*		x			Guaraciama	Free-living	Female	Juvenile	negative
Minas Gerais	MG143	*Callithrix penicillata*		x			Juramento	Free-living	Female	Adult	negative
Minas Gerais	MG144	*Callithrix penicillata*		x			São João da Lagoa	Free-living	Female	Juvenile	negative
Minas Gerais	MG146	*Callithrix penicillata*		x			São João da Lagoa	Free-living	Female	Juvenile	negative
Minas Gerais	MG148	*Callithrix penicillata*		x			Francisco Sá	Free-living	Female	Adult	negative
Minas Gerais	MG149	*Callithrix penicillata*		x			Francisco Sá	Free-living	Male	Adult	negative
Minas Gerais	MG150	*Callithrix penicillata*		x			São João da Lagoa	Free-living	Male	Juvenile	negative
Minas Gerais	MG151	*Callithrix penicillata*		x			São João da Lagoa	Free-living	Male	Juvenile	negative
Minas Gerais	MG152	*Callithrix penicillata*		x			São João do Pacuí	Free-living	Male	Adult	negative
Minas Gerais	MG153	*Callithrix penicillata*		x			São João do Pacuí	Free-living	Female	Juvenile	negative
Minas Gerais	MG154	*Callithrix penicillata*		x			São João do Pacuí	Free-living	Male	Juvenile	negative
Minas Gerais	MG155	*Callithrix penicillata*		x			São João da Lagoa	Free-living	Female	Juvenile	negative
Minas Gerais	MG156	*Callithrix penicillata*		x			Jequitaí	Free-living	Female	Adult	negative
Minas Gerais	MG158	*Callithrix penicillata*		x			São João da Lagoa	Free-living	Female	Baby	negative
Minas Gerais	MG161	*Alouatta caraya*		x	x		Salinas	Free-living	Male	Adult	negative

Minas Gerais	MG162	*Callithrix penicillata*		x			Salinas	Free-living	Male	Juvenile	negative
Minas Gerais	MG163	*Callithrix penicillata*	x				Salinas	Free-living	Male	Adult	negative
Minas Gerais	MG164	*Callithrix penicillata*	x				Salinas	Free-living	Female	Adult	negative
Minas Gerais	MG166	*Callithrix penicillata*	x				Bonito de Minas	Free-living	Male	Adult	negative
Minas Gerais	MG167	*Callithrix penicillata*	x				Bonito de Minas	Free-living	Male	Adult	negative
Minas Gerais	MG168	*Callithrix penicillata*	x				Bonito de Minas	Free-living	Male	Adult	negative
Minas Gerais	MG169	*Callithrix geoffroyi*	x				Teófilo Otoni	Free-living	Female	Adult	negative
Minas Gerais	MG170	*Callithrix geoffroyi*	x				Teófilo Otoni	Free-living	Female	Adult	negative
Minas Gerais	MG171	*Callithrix geoffroyi*	x				Teófilo Otoni	Free-living	Male	Adult	negative
Minas Gerais	MG172	*Callithrix geoffroyi*	x				Teófilo Otoni	Free-living	Female	Adult	negative
Minas Gerais	MG173	*spp. (hibrid C. penicillata /*	x				Almenara	Free-living	Female	Juvenile	negative
Minas Gerais	MG174	*spp. (hibrid C. penicillata /*	x				Almenara	Free-living	Female	Adult	negative
Minas Gerais	MG175	*spp. (hibrid C. penicillata /*	x				Almenara	Free-living	Female	Adult	negative
Minas Gerais	MG176	*spp. (hibrid C. penicillata /*	x				Almenara	Free-living	Female	Juvenile	negative
Minas Gerais	MG177	*spp. (hibrid C. penicillata /*	x				Almenara	Free-living	Male	Juvenile	negative
Minas Gerais	MG180	*Callithrix penicillata*		x			Salinas	Free-living	Female	Adult	negative
Minas Gerais	MG182	*Callithrix penicillata*		x			Salinas	Free-living	Female	Juvenile	negative
Minas Gerais	MG183	*Callithrix penicillata*		x			Salinas	Free-living	Male	Adult	negative
Minas Gerais	MG185	*Callithrix penicillata*		x			Salinas	Free-living	Female	Adult	negative
Minas Gerais	MG186	*Callithrix penicillata*		x			Araçuaí	Free-living	Female	Adult	negative
Minas Gerais	MG187	*Callithrix penicillata*		x			Salinas	Free-living	Male	Adult	negative
Minas Gerais	MG188	*Alouatta caraya*		x			Salinas	Free-living	Male	Adult	negative
Minas Gerais	MG189	*Callithrix penicillata*		x			Salinas	Free-living	Female	Juvenile	negative
Minas Gerais	MG190	*Callicebus melanochir*		x			Monte Formoso	Free-living	Male	Adult	negative
Minas Gerais	MG191	*Alouatta caraya*		x			Ubaí	Free-living	Female	Adult	negative
Minas Gerais	MG192	*Alouatta caraya*		x			Ubaí	Free-living	Female	Adult	negative
Minas Gerais	MG193	*Sapajus xanthosternos*		x			Montes Claros	Free-living	Male	Adult	negative
Bahia	A.01	*Alouatta caraya*	x				Cocos	Free-living	Female	Juvenile	negative
Bahia	A.02	*Alouatta caraya*	x				Santa Rita de Cássia	Free-living	Male	Adult	negative

Bahia	C.01	*Callithrix penicillata*	x				Cocos	Free-living	Male	Adult	negative
Bahia	C.02	*Callithrix penicillata*	x				Cocos	Free-living	Male	Adult	negative
Bahia	C.03	*Callithrix penicillata*	x				Cocos	Free-living	Male	Adult	negative
Bahia	C.04	*Callithrix penicillata*	x				Cocos	Free-living	Female	Adult	negative
Bahia	C.07	*Callithrix penicillata*	x				Coribe	Free-living	Female	Adult	negative
Bahia	C.08	*Callithrix penicillata*	x				Coribe	Free-living	Male	Adult	negative
Bahia	C.09	*Callithrix penicillata*	x				Coribe	Free-living	Male	Adult	negative
Bahia	C.10	*Callithrix penicillata*	x				Coribe	Free-living	Male	Adult	negative
Bahia	C.20	*Callithrix penicillata*	x				Santa Rita de Cássia	Free-living	Male	Adult	negative
Bahia	C.21	*Callithrix penicillata*	x				Santa Rita de Cássia	Free-living	Male	Adult	negative
Bahia	C.22	*spp. (hibrid C. penicillata /*	x				Santa Rita de Cássia	Free-living	Male	Baby	negative
Bahia	C.23	*spp. (hibrid C. penicillata /*	x				Santa Rita de Cássia	Free-living	Female	Adult	negative
Bahia	C.24	*spp. (hibrid C. penicillata /*	x				Santa Rita de Cássia	Free-living	Male	Adult	negative
Bahia	C.25	*Callithrix jacchus*	x				Santa Rita de Cássia	Free-living	Male	Adult	negative
Bahia	C.26	*Callithrix jacchus*	x				Santa Rita de Cássia	Free-living	Female	Juvenile	negative
Bahia	C.27	*Callithrix jacchus*	x				Santa Rita de Cássia	Free-living	Male	Adult	negative
Bahia	C.28	*spp. (hibrid C. penicillata /*	x				Santa Rita de Cássia	Free-living	Female	Adult	negative
Bahia	C.29	*spp. (hibrid C. penicillata /*	x				Santa Rita de Cássia	Free-living	Female	Juvenile	negative
Bahia	C.31	*Callithrix penicillata*	x				Formosa do Rio Preto	Free-living	Male	Adult	negative
Bahia	C.32	*Callithrix penicillata*	x				Formosa do Rio Preto	Free-living	Female	Adult	negative
Bahia	C.33	*Callithrix penicillata*	x				Formosa do Rio Preto	Free-living	Female	Adult	negative
Bahia	C.34	*Callithrix penicillata*	x				Formosa do Rio Preto	Free-living	Male	Adult	negative
Bahia	C.35	*Callithrix jacchus*	x				Formosa do Rio Preto	Free-living	Male	Baby	negative
Bahia	C.36	*spp. (hibrid C. penicillata /*	x				Formosa do Rio Preto	Free-living	Female	Adult	negative
Bahia	C.37	*spp. (hibrid C. penicillata /*	x				Formosa do Rio Preto	Free-living	Female	Adult	negative
Bahia	C.38	*spp. (hibrid C. penicillata /*	x				Formosa do Rio Preto	Free-living	Male	Adult	negative
Bahia	C.39	*spp. (hibrid C. penicillata /*	x				Formosa do Rio Preto	Free-living	Male	Adult	negative
Bahia	C.40	*Callithrix geoffroyi*	x				Itanhém	Free-living	Male	Adult	negative
Bahia	C.41	*Callithrix geoffroyi*	x				Itanhém	Free-living	Male	Adult	negative

Bahia	C.42	*Callithrix geoffroyi*	x				Itanhém	Free-living	Male	Juvenile	negative
Bahia	C.43	*Callithrix geoffroyi*	x				Itanhém	Free-living	Female	Adult	negative
Bahia	C.44	*Callithrix geoffroyi*	x				Itanhém	Free-living	Female	Adult	negative
Bahia	C.46	*Callithrix geoffroyi*				x	Porto Serguro	Free-living	Male	Adult	positive
Bahia	C.47	*Callithrix geoffroyi*	x				Porto Serguro	Free-living	Female	Adult	negative
Bahia	C.48	*Callithrix geoffroyi*	x				Porto Serguro	Free-living	Female	Juvenile	negative
Bahia	C.49	*Callithrix geoffroyi*				x	Porto Serguro	Free-living	Male	Adult	negative
Bahia	C.50	*Callithrix geoffroyi*	x				Porto Serguro	Free-living	Female	Adult	negative
Bahia	C.51	*Callithrix geoffroyi*	x				Porto Serguro	Free-living	Female	Adult	negative
Bahia	C.52	*Callithrix kuhlli*	x				Ilhéus	Free-living	Female	Adult	negative
Bahia	C.53	*Callithrix kuhlli*	x				Ilhéus	Free-living	Female	Adult	negative
Bahia	C.54	*Callithrix kuhlli*	x				Ilhéus	Free-living	Male	Juvenile	negative
Bahia	C.55	*Callithrix kuhlli*	x				Ilhéus	Free-living	Male	Juvenile	negative
Bahia	C.56	*Callithrix kuhlli*	x				Ilhéus	Free-living	Male	Adult	negative
Bahia	C.57	*Callithrix kuhlli*	x				Ilhéus	Free-living	Male	Adult	negative
Bahia	C.58	*Callithrix kuhlli*				x	Ilhéus	Free-living	Male	Adult	negative
Bahia	C.59	*Callithrix kuhlli*	x				Ilhéus	Free-living	Male	Adult	negative
Bahia	C.60	*Callithrix kuhlli*				x	Ilhéus	Free-living	Female	Adult	negative
Bahia	C.61	*Callithrix kuhlli*				x	Ilhéus	Free-living	Female	Adult	negative
Bahia	C.62	*Callithrix kuhlli*				x	Ilhéus	Free-living	Female	Adult	negative
Bahia	C.63	*Callithrix kuhlli*				x	Ilhéus	Free-living	Male	Juvenile	negative
Bahia	C.64	*Callithrix kuhlli*				x	Ilhéus	Free-living	Male	Adult	negative
Bahia	C.66	*Callithrix kuhlli*				x	Ilhéus	Free-living	Male	Juvenile	negative
Bahia	C.67	*Callithrix kuhlli*				x	Ilhéus	Free-living	Male	Juvenile	negative
Bahia	C.68	*Callithrix kuhlli*				x	Ilhéus	Free-living	Male	Adult	negative
Bahia	C.70	*Callithrix kuhlli*				x	Ilhéus	Free-living	Female	Juvenile	negative
Bahia	C.71	*Callithrix kuhlli*				x	Ilhéus	Free-living	Female	Adult	negative
Bahia	C.72	*Callithrix kuhlli*				x	Ilhéus	Free-living	Male	Adult	negative
Bahia	C.73	*Callithrix kuhlli*				x	Ilhéus	Free-living	Male	Adult	negative

Bahia	C.74	*Callithrix kuhlli*				x	Ilhéus	Free-living	Male	Juvenile	negative
Bahia	C.76	*Callithrix kuhlli*				x	Ilhéus	Free-living	Female	Juvenile	negative
Bahia	C.78	*Callithrix kuhlli*				x	Ilhéus	Free-living	Male	Adult	negative
Bahia	C.80	*Callithrix kuhlli*				x	Ilhéus	Captive	Female	Adult	negative
Bahia	C.83	*Callithrix kuhlli*				x	Ilhéus	Free-living	Female	Baby	negative
Bahia	C.84	*Callithrix kuhlli*				x	Ilhéus	Free-living	Male	Adult	negative
Bahia	C.85	*Callithrix kuhlli*				x	Ilhéus	Free-living	Male	Adult	negative
Bahia	C.100	*Callithrix penicillata*	x				Malhada	Free-living	Male	Adult	negative
Bahia	C.101	*Callithrix penicillata*	x				Iuiú	Free-living	Male	Juvenile	negative
Bahia	C.102	*Callithrix penicillata*	x				Iuiú	Free-living	Female	Juvenile	negative
Bahia	C.103	*Callithrix penicillata*	x				Iuiú	Free-living	Male	Adult	negative
Bahia	C.104	*Callithrix penicillata*	x				Iuiú	Free-living	Female	Adult	negative
Bahia	C.105	*Callithrix penicillata*	x				Carinhanha	Free-living	Female	Adult	negative
Bahia	C.106	*Callithrix penicillata*	x				Carinhanha	Free-living	Male	Adult	negative
Bahia	C.107	*Callithrix penicillata*	x				Cocos	Free-living	Male	Adult	negative
Bahia	C.108	*Callithrix penicillata*	x				Cocos	Free-living	Female	Adult	negative
Bahia	C.109	*Callithrix penicillata*	x				Cocos	Free-living	Female	Adult	negative
Bahia	C.110	*Callithrix penicillata*	x				Cocos	Free-living	Male	Adult	negative
Bahia	C.114	*Callithrix penicillata*	x				Coribe	Free-living	Female	Adult	negative
Bahia	C.115	*Callithrix penicillata*	x				Coribe	Free-living	Male	Adult	negative
Bahia	C.116	*Callithrix penicillata*	x				Coribe	Free-living	Male	Adult	negative
Bahia	C.117	*Callithrix penicillata*	x				Coribe	Free-living	Female	Adult	negative
Bahia	C.118	*Callithrix penicillata*	x				Coribe	Free-living	Male	Adult	negative
Bahia	C.119	*Callithrix penicillata*	x				Coribe	Free-living	Male	Adult	negative
Bahia	C.120	*Callithrix penicillata*	x				Coribe	Free-living	Female	Adult	negative
Bahia	C.122	*Callithrix penicillata*	x				Jaborandi	Free-living	Female	Adult	negative
Bahia	C.123	*Callithrix penicillata*	x				Jaborandi	Free-living	Male	Adult	negative
Bahia	C.124	*Callithrix penicillata*	x				Jaborandi	Free-living	Female	Adult	negative
Bahia	C.127	*Callithrix penicillata*	x				Feira de Santana	Free-living	Male	Adult	negative

Bahia	C.128	*Callithrix penicillata*	x				Cruz das Almas	Captive	Male	Adult	negative
Bahia	C.129	*[illegible] spp. (hibrid C. penicillata / [illegible]*	x				Cruz das Almas	Captive	Female	Juvenile	negative
Bahia	C.140	*Callithrix geoffroyi*	x				Itabela	Free-living	Female	Adult	negative
Bahia	C.141	*Callithrix geoffroyi*	x				Itabela	Free-living	Female	Adult	negative
Bahia	C.143	*Callithrix geoffroyi*	x				Porto Serguro	Free-living	Male	Adult	negative
Bahia	C.145	*Callithrix geoffroyi*	x				Porto Serguro	Free-living	Female	Baby	negative
Bahia	C.146	*Callithrix geoffroyi*	x				Porto Serguro	Free-living	Male	Adult	negative
Bahia	C.147	*Callithrix geoffroyi*	x				Porto Serguro	Free-living	Female	Adult	negative
Bahia	C.149	*Callithrix geoffroyi*	x				Porto Serguro	Free-living	Male	Adult	negative
Bahia	C.150	*Callithrix geoffroyi*	x				Porto Serguro	Free-living	Female	Adult	negative
Bahia	C.151/C.5[illegible]	*Callithrix geoffroyi*	x				Porto Serguro	Free-living	Female	Adult	negative
Bahia	C.152/C.4[illegible]	*Callithrix geoffroyi*	x				Porto Serguro	Free-living	Female	Adult	negative
Bahia	C.153	*Callithrix geoffroyi*	x				Porto Serguro	Free-living	Female	Adult	negative
Bahia	C.154	*Callithrix geoffroyi*	x				Porto Serguro	Free-living	Female	Juvenile	negative
Bahia	C.155	*Callithrix kuhlli*	x				Ilhéus	Free-living	Female	Baby	negative
Bahia	C.156	*Callithrix kuhlli*	x				Ilhéus	Free-living	Female	Adult	negative
Bahia	C.157	*Callithrix kuhlli*	x				Ilhéus	Free-living	Male	Baby	negative
Bahia	C.158	*Callithrix kuhlli*	x				Ilhéus	Free-living	Male	Adult	negative
Bahia	C.198	*Callithrix penicillata*	x				Lençóis	Free-living	Male	Adult	negative
Bahia	C.199	*Callithrix penicillata*	x				Lençóis	Free-living	Female	Juvenile	negative
Bahia	C.200	*Callithrix penicillata*	x				Lençóis	Free-living	Female	Adult	negative
Bahia	L.01	*Leontopithecus chrysomelas*				x	Ilhéus	Free-living	Female	Adult	negative
Bahia	L.02	*Leontopithecus chrysomelas*				x	Ilhéus	Free-living	Male	Adult	negative
Bahia	L.03	*Leontopithecus chrysomelas*				x	Ilhéus	Free-living	Male	Adult	negative
Bahia	L.04	*Leontopithecus chrysomelas*	x				Ilhéus	Free-living	Male	Adult	negative
Bahia	L.05	*Leontopithecus chrysomelas*	x				Ilhéus	Free-living	Male	Adult	negative
Bahia	L.06	*Leontopithecus chrysomelas*				x	Ilhéus	Free-living	Male	Adult	negative
Bahia	L.07	*Leontopithecus chrysomelas*				x	Ilhéus	Free-living	Male	Juvenile	negative
Bahia	L.16	*Leontopithecus chrysomelas*				x	Una	Free-living	Male	Adult	negative

Bahia	L.17	*Leontopithecus chrysomelas*	x				Una	Free-living	Female	Adult	negative
Bahia	L.18	*Leontopithecus chrysomelas*	x				Una	Free-living	Male	Adult	negative
Bahia	L.19	*Leontopithecus chrysomelas*	x				Una	Free-living	Female	Adult	negative
Bahia	L.20	*Leontopithecus chrysomelas*	x				Una	Free-living	Female	Adult	negative
Bahia	L.21	*Leontopithecus chrysomelas*	x				Una	Free-living	Male	Adult	negative
Bahia	L.22	*Leontopithecus chrysomelas*	x				Una	Free-living	Male	Adult	negative
Bahia	L.24	*Leontopithecus chrysomelas*	x				Una	Free-living	Male	Adult	negative
Bahia	L.25	*Leontopithecus chrysomelas*	x				Una	Free-living	Male	Adult	negative
Bahia	S.01	*Sapajus robustus*	x				Jucuruçu	Captive	Male	Adult	negative
